# A Retrospective Cohort Study to Investigate the Impact of Cardiovascular Comorbidities on Knee Osteoarthritis Progression

**DOI:** 10.31138/mjr.34.2.279

**Published:** 2023-03-30

**Authors:** Hugo Parente, Francisca Guimarães, Catarina Dantas Soares, Maria Pontes Ferreira, Soraia Azevedo, Daniela Santos-Faria, Daniela Peixoto, Carmo Afonso, José Tavares-Costa, Filipa Teixeira

**Affiliations:** Rheumatology Department, Unidade Local de Saúde do Alto Minho, Ponte de Lima, Portugal

**Keywords:** knee osteoarthritis, cardiovascular risk factors, meta-inflammation, total knee arthroplasty

Dear Editor,

Osteoarthritis (OA) and cardiovascular (CV) diseases are paramount conditions in developed countries, contributing widely to the global health burden.^1^ OA patients are more likely to have metabolic/CV comorbidities, which affect OA progression,^2^ and meta-inflammation may be the underlying connection.^3^ However, on knee OA, risk factors for progression are mainly biomechanical imbalances.^4^ Obesity, as both an OA and CV risk factor, 5 is a confounding component. Sarcopenia, sedentary lifestyle, and subchondral ischemia from compromised circulation further convolute this affiliation.

To determine the impact of CV comorbidities on knee OA, based on radiographic rate of progression (using the Kellgren-Lawrence’s [KL] severity OA scale) and the need for total knee arthroplasty (TKA), we designed a cohort-retrospective study among non-obese primary knee OA patients, at a Portuguese hospital, from 2017–2020. Sociodemographic and clinical data were collected, and both cohorts, with and without CV risk factors, were compared using SPSSv25.

A total of 304 patients were eligible, characterized in **Table 1**. CV risk factors were associated with higher KL score at baseline [OR=2.118 (CI 95%:1.264–3.549)] and 5 years later [OR=14.960 (CI 95%:3.439–65.079)], faster (2-grade worsening) KL progression [OR=2.755 (CI 95%:1.205–6.299)], and greater TKA need [OR=3.781 (CI 95%:2.257–6.335)]. The number of CV risk factors did not compound worse KL progression or TKA need. Essential Arterial Hypertension was associated with higher KL score, both at baseline [OR=1.819 (CI 95%:1.108–2.987)] and 5 years later [OR=17.921 (CI 95%:2.382–134.835)], but not with faster KL progression. It was also associated with a greater TKA need [OR=3.320 (CI 95%:1.197–5.750)]. Dyslipidaemia was associated with higher KL score at baseline [OR=1.843 (CI 95%: 1.120–3.030)] and 5 years later [OR=7.709 (CI 95%: 1.772–33.530)], faster KL progression [OR=2.560 (CI 95%:1.249–5.246)], and greater TKA need [OR=2.493 (CI 95%:1.457–4.268)]. Type-2 Diabetes Mellitus was associated with higher KL score at the 5-year mark [OR=1.103 (CI 95%:1.059–1.148)], but not with other measures. Evaluated medications showed no association with other variables.

**Table 1.** Cohort stratified characteristics.

	**Total** (n=304)	**With CV risk factors** (n=169)	**Without CV risk factors** (n=135)	**p-value**

Age at diagnosis (years), Mean (SD)	73.09 (8.74)	74.47 (7.67)	71.36 (9.67)	0.002

Gender, female, % (n/N)	71.7 (218/304)	71.6 (121/169)	71.9 (97/135)	NS

CV risk factors prior to OA diagnosis				
- Essential Arterial Hypertension, % (n/N)	76.4 (129/169)	76.4 (129/169)	-	
- Dyslipidaemia, % (n/N)	72.2 (122/169)	72.2 (122/169)	-	-
- Type-2 Diabetes Mellitus, % (n/N)	30.8 (52/169)	30.8 (52/169)	-	

Number of CVRF				
- 0, % (n/N)	44.4 (135/304)	-	100 (135/135)	
- 1, % (n/N)	18.8 (57/304)	33.7 (57/169)	-	
- 2, % (n/N)	29.6 (90/304)	53.3 (90/169)	-	-
- 3, % (n/N)	7.2 (22/304)	13 (22/169)	-	

Medication history				
- Fenofibrate, % (n/N)	3 (5/169)	3 (5/169)	-	
- Statin, % (n/N)	67.5 (114/169)	67.5 (114/169)	-	-
- Metformin, % (n/N)	23.7 (40/169)	23.7 (40/169)	-	
- Liraglutide, % (n/N)	2.4 (4/169	2.4 (4/169 )	-	

Radiographic scoring through KL classification system				
At baseline				0.006
- 2, % (n/N)	70.1 (213/304)	63.3 (107/169)*	78.5 (106/135)*	*−2.9, 2.9
- 3, % (n/N)	28.9 (88/304)	34.9 (59/169)*	21.5 (29/135)*	*2.6, −2.6
- 4, % (n/N)	1.0 (3/304)	1.8 (3/169)	-	
After 5 years				<0.001
- 2, % (n/N)	7.6 (23/304)	1.2 (2/169)*	15.7 (21/135)*	*−4.6, 4.6
- 3, % (n/N)	53.9 (164/304)	47.9 (78/169)*	64.2 (86/135)*	*−2.8, 2.8
- 4, % (n/N)	36.2 (110/304)	50.9 (83/169)*	20.1 (27/135)*	*5.5, −5.5

KL progression				<0.001
- none, % (n/N)	15.5 (47/304)	8.9 (15/169)*	23.7 (32/135)*	*−3.6, 3.6
- 1 stage, % (n/N)	72.4 (220/304)	74.0 (125/169)	70.4 (95/135)	
- 2 stages, % (n/N)	12.2 (37/304)	17.1 (29/169)*	5.9 (8/135)*	*3.0, −3.0

Need for TKA, yes, % (n/N)	69.4 (211/304)	81.7 (138/169)	54.1 (73/135)	<0.001

Time to TKA, Mdn (AIQ)	6 (4)	6 (4)	6 (3)	NS

* Adjusted residue values – positive association if > 1,96 and negative if < −1.96.

CV: cardiovascular; IQR: interquartile range; KL: Kellgren-Lawrence; Mdn: median; NS: non-significant; OA: osteoarthritis; TKA: total knee arthroplasty.

In conclusion, CV risk factors contribute to OA progression. Calvet et al.^6^ had already found, in a 254-patient control-based study, a higher frequency of Metabolic Syndrome and CV events in symptomatic knee and hand OA patients. In this logic, a “metabolic OA” subtype has been proposed,^7^ noting that OA is not just age- or weight-related. In our study, Essential Arterial Hypertension and Dyslipidaemia patients showed worse radiographic OA and greater TKA need, with Dyslipidaemia speeding up KL progression. This latter association is supported by a previous meta-analysis.^8^ Type-2 Diabetes Mellitus’ role might, nonetheless, be dependent on obesity, as stated by others.^6,9^ Having more than one CV risk factor was not associated with an increased disease progression, and cardiovascular therapeutic medications did not award an overall protective role. In this regard, a robust meta-analysis is lacking, but a review on the effect of statins in OA^10^ has found a significant decreased risk of OA in statin-users, as well as a lessened radiographic progression, accompanied by a small symptomatic improvement. Patient adherence, duration of treatment and cumulative doses might explain the discrepancies among studies. While limited by its observational design and possible confounding factors, this study bolsters the integrated management of CV risk factors in knee OA patients, as a potential mitigation to structural progression. Given the expected progressive ageing of the population in the coming years, these conditions (OA and CV risk factors) should further sustain their relation.
